# Draft *de novo* genome construction of *Scytonema* sp. PRP1: identified from single-cell sequencing library preparation

**DOI:** 10.1128/mra.00029-25

**Published:** 2025-07-31

**Authors:** Preston Parana, Camille Mumm, Michael J. McConnell, Alan P. Boyle

**Affiliations:** 1 Department of Human Genetics, University of Michigan 1259, Ann Arbor, Michigan, USA; 2 Lieber Institute for Brain Development 466099, Baltimore, Maryland, USA; 3 Department of Computational Medicine and Bioinformatics, University of Michigan 1259, Ann Arbor, Michigan, USA; The University of Arizona, Tucson, Arizona, USA

**Keywords:** genome, cyanobacteria, nanopore, gene sequencing, DNA sequencing

## Abstract

We present the genome sequence of *Scytonema* sp. PRP1, a cyanobacterium identified during single-cell sequencing library preparation of de-identified human brain samples associated with the Brain Somatic Mosaicism Network. Our 8,266,022 bp genome comprises 140 contigs, containing 6,765 protein-coding sequences, 37 tRNA genes, and 11 rRNA genes.

## ANNOUNCEMENT

*Scytonema sp*. PRP1 was identified as a contaminant during single-cell sequencing library preparation of de-identified human brain samples (1). A single brain nucleus preparation was amplified using multiple annealing and looping-based amplification cycles (MALBAC) at the University of Virginia School of Medicine per Burbulis et al. (2). Although the contamination source remains uncertain, shared buffers were consistently used during the preparation and sequencing of >100 nuclei from the same sample (3), making reagent contamination unlikely. Given MALBAC amplification’s high sensitivity, circulating environmental spores are a likely source of this species.

**Fig 1:**
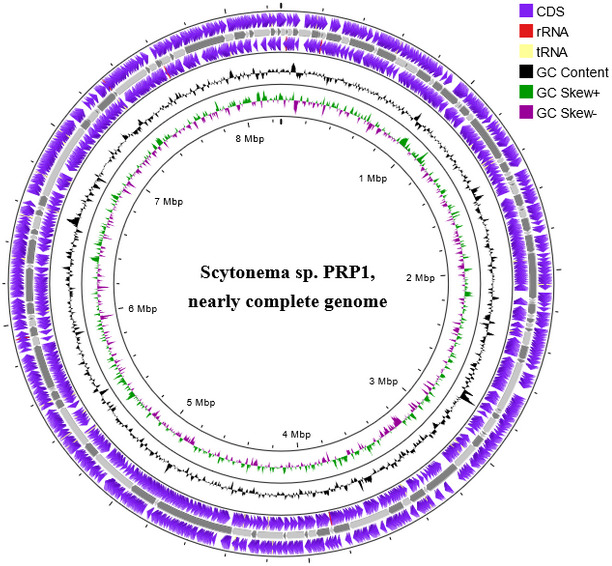
Circular genome of* Scytonema* sp. PRP1 created via Proksee, indicating (outer to inner) CDS on the forward and reverse strands (indigo), tRNAs (yellow), rRNAs (red), GC content (black), positive GC skew (green), and negative GC skew (purple).

As previously described in Burbulis et al. (2), cells were lysed using 2.5 µL buffer (50 mM Tris pH 8.8, 50 mM NaCl, 50 mM KCl, 1 µM EDTA, 1 µM DTT, 0.1% Tween-20, and 20 mg/mL Proteinase K [Qiagen]), digested at 55°C for 3 h, then inactivated at 75°C and 80°C. Lysates were supplemented with amplification buffer (3 mM dNTPs, 0.667 µM primers) and underwent 5 MALBAC heating/cooling cycles, with enzyme mix added at each cycle. After PCR amplification, DNA was precipitated with NaCl and ethanol (2), purified via centrifugation and QIAquick PCR columns, quantified using Qubit 2.0 HS DNA assay, and prepared for sequencing. Libraries of five single-cell MALBAC-amplified products were prepared with 300 ng barcoded-adapted sample (ONT Native Barcoding kit 24 V14, SQK-NBD114.24) and sequenced on a MinION R10.4.1 flow cell (Mk1B). Genomic DNA was neither fragmented nor size-selected. Data were basecalled using Guppy v6.2.11 (ONT, score filter 9). Chimeric reads were split using duplex_tools (ONT), and adapter trimmed using Porechop v0.2.4 (4). This sample yielded 1,226,304 passing reads; after removing reads that mapped to the human genome (3.3%), 1,185,629 remained for assembly (N50 = 1,414 bp), of which 86.43% mapped to the *Scytonema* genus using Blobtools v1.1.1 (5) and BLASTn v2.14.0 (6). Unicycler v0.5.0 (7) was used for hybrid genome assembly in bold mode; all software used default parameters unless otherwise noted. Contigs <400 bp were filtered (5), and Medaka v1.11.1 (8) polished the final assembly, producing a 101.7× coverage genome of 8.26 Mb with 140 contigs, an N50 of 107.9 KB, a completeness of 82.2%, and 43.5% GC content. QUAST v5.2.0 (9) and BUSCO v5.5.0 (10) assessed genome contiguity and completeness. Sequences were submitted to GenBank and annotated using PGAP v6.6 (11), predicting 6,765 protein-coding genes, 7 CRISPR genes, 11 rRNA genes, 37 tRNA genes, 395 pseudogenes, and 4 ncRNA genes. Proksee (https://proksee.ca) visualized the annotated genome (Fig. 1).

GTDB-Tk v2.1.1 (12) assigned PRP1 to the *Scytonema* genus, identifying *Scytonema sp*. NIES-4073 (GenBank accession AP018268.1) as its closest relative (FastANI: 95.8%). This is supported by the whole genome phylogenetic tree generated by the Type (Strain) Genome Server (TYGS) (13), suggesting PRP1 and NIES-4073 are distinct species originating from the same common ancestor.

## Data Availability

The whole genome shotgun project of *Scytonema* sp. PRP1 has been deposited in GenBank under the accession no. JBAHYJ000000000, and the raw data has been deposited under the accession no. SRR27967422.
